# Bioactive Ingredients in *K. pinnata* Extract and Synergistic Effects of Combined *K. pinnata* and Metformin Preparations on Antioxidant Activities in Diabetic and Non-Diabetic Skeletal Muscle Cells

**DOI:** 10.3390/ijms24076211

**Published:** 2023-03-25

**Authors:** Pedro Ramon, Daniela Bergmann, Hussain Abdulla, Jean Sparks, Felix Omoruyi

**Affiliations:** 1 Department of Physical and Environmental Sciences, Texas A&M University, Corpus Christi, TX 78412, USA; 2 Department of Health Sciences, Texas A&M University, Corpus Christi, TX 78412, USA; 3 Department of Life Sciences, Texas A&M University, Corpus Christi, TX 78412, USA

**Keywords:** *K. pinnata*, diabetic skeletal muscle cells, oxidative stress, type II diabetes mellitus

## Abstract

With healthcare costs rising, many affected by ailments are turning to alternative medicine for treatment. More people are choosing to complement their pharmacological regimen with dietary supplements from natural products. In this study, the compound composition of *Kalanchoe Pinnata* (*K. pinnata)* and the effects of combined preparations of *K. pinnata* and metformin on antioxidant activity in human skeletal muscle myoblasts (HSMMs) and human diabetic skeletal muscle myoblasts (DHSMMs) were investigated. Ultraperformance liquid chromatography fusion orbitrap mass spectrometry (UPLC-OT-FTMS) identified biologically active flavanols in *K. pinnata*. The main compounds identified in locally grown *K. pinnata* were quercetin, kaempferol, apigenin, epigallocatechin gallate (EGCG), and avicularin. Antioxidant results indicated that a combinatorial preparation of *K. pinnata* with metformin may modulate antioxidant responses by increasing the enzymatic activity of superoxide dismutase and increasing levels of reduced glutathione. A combination of 50 μM and 150 μg/mL of metformin and *K. pinnata*, respectively, resulted in a significant increase in reduced glutathione levels in non-diabetic and diabetic human skeletal muscle myoblasts and H_2_O_2-_stress-induced human skeletal muscle myoblasts. Additionally, a *K. pinnata* treatment (400 µg/mL) alone significantly increased catalase (CAT) activity for non-diabetic and diabetic human skeletal muscle myoblasts and a H_2_O_2-_stress-induced human skeletal muscle myoblast cell line, while significantly lowering malondialdehyde (MDA) levels. However, the treatment options were more effective at promoting cell viability after 24 h versus 72 h and did not promote cell viability after 72 h in H_2_O_2_-stress-induced HSMM cells. These treatment options show promise for treating oxidative-stress-mediated pathophysiological complications associated with type II diabetes.

## 1. Introduction

Diabetes mellitus, more simply known as “diabetes,” is a chronic disease identified by abnormally high levels of glucose in the bloodstream [1]. Type II diabetic patients do not respond to the increasing concentration of glucose in the blood after a meal [1]. When glucose levels become significantly high, the risk of health complications significantly increases [1,2]. Diabetes has the potential to damage organs such as the eyes, kidneys, and nerves [1]. As high glucose concentration builds up in the blood of diabetic patients, the rate of oxidative stress in cells increases, forcing them to secrete pro-inflammatory mediators in response [3]. In a hyperglycemic state, superoxide anion production increases [4]. If cellular oxidation occurs at a fast rate, free radicals will begin to accumulate in the bloodstream. The free radicals are capable of damaging proteins and nucleic acids that are vital to cellular function. Matough et al. demonstrated that type II diabetes induces the generation of free radicals while suppressing cellular antioxidant defenses [5]. Kawahito et al. reported that oxidative damage leads to further diabetic complications in those affected by the disease [3].

There are various classes of drugs that lower blood glucose levels in type II diabetes. However, metformin, along with physical activity, is usually the first combinatorial treatment offered by physicians [6]. As healthcare costs continue to rise, many are turning to alternative medicines for treatment and are combining them with modern medicine.

*Kalanchoe pinnata* is a plant native to the Caribbean, African, and South American regions, and the leaves and stems of the plant are used for the treatment of various diseases and ailments [7,8]. *Kalanchoe pinnata*’s potential as a herbal treatment stems from the fact that there is a wide range of active compounds reported in the leaves and roots of the plant [9]. Alkaloids, glycosides, flavonoids, steroids, bufadienolides, lipids, and organic acids have been identified in pharmacological studies, where these compounds have acted as immunomodulators, CNS depressants, and analgesic, antimicrobial, anti-inflammatory, antifungal, and anticancerous treatments [9]. In a study where the chemical constituents of *K. pinnata*’s leaves were analyzed by gas chromatography-mass spectrometry (GC-MS), 5-Dihydroxy-6-methyl-2, 3-dihydro-4H-pyran-4-one (DDMP) and Alpha-d-glucopyranoside were detected [10]. These compounds have been reported to have antituberculosis, antioxidant, anticonvulsant, and alpha-amylase inhibitory activity [11,12,13]. In another study, nine other phytochemicals were identified by GC-MS, confirming that biologically active compounds are concentrated within *K. pinnata*’s leaves [10]. The presence of various phytochemicals with various biological properties rationalizes the use of *K. pinnata* leaf extracts in folklore medicine. Despite *K. pinnata* being utilized in pharmacological studies, *K. pinnata*’s use in the treatment of diabetes and diabetic inflammation has been limited in the literature [7]. We have previously reported that the combined preparations of metformin and *K. pinnata* may modulate immune responses that could potentially promote inflammation in HSMM and DHSMM cells [14]. In that study, there was a significant contrast in cellular viability between HSMM cells and stress-induced HSMM cells and a significant difference in cell viability between HSMM and DHSMM cells (Table 1). We also noted significant changes in the effects of the treatment options among the cell types after 72 h of treatment, indicating that treatment options were unaffected by cell type after 24 h. We reported that the difference might be due to oxidative damage induced by a type II diabetes disease state. It was also noted that the DHSMM cells were five times more likely to survive than HSMM cells after 72 h post-treatment and suggested that a combinatorial *K. pinnata* and metformin preparation may protect DHSMMs from oxidative stress. However, the H_2_O_2_-stress-induced HSMM cell viability was reduced after 72 h among the treatment groups. It was, however, unclear why there was an increase in DHSMM viability and HSMM viability after treatment with metformin only, *K. pinnata* only, and different combinations of metformin and *K. pinnata* after 72 h versus lower cell viability on day 1.

Although the combined preparations of metformin and *K. pinnata* may modulate immune responses that may promote inflammation, it was not clear whether the increase in cell viability may be associated with the counter effects of the antioxidant activity of *K. pinnata* that overall minimize the impacts of the reported increased concentration of inflammatory markers. Antioxidants within *K. pinnata* have been shown to affect cellular proliferation, apoptosis, and autophagy and modulate immune responses [15,16].

Because antioxidants influence many cell functions, we investigated whether a *K. pinnata* combinatorial treatment with metformin would benefit diabetic human muscle cells. Therefore, we explored various antioxidant markers to determine whether antioxidant concentrations correlated to increased cellular viability.

In addition, the bioactive components of locally grown *K. pinnata* and the effects of metformin or *K. pinnata* or the combination treatment on induced oxidative stress in human skeletal muscle myoblasts and diabetic skeletal muscle myoblasts are unknown. Hence, this study also investigated the compound composition of locally (Corpus Christi, TX, USA) grown *K. pinnata*. Human skeletal muscle myoblast (HSMM), oxidative-stress-induced human skeletal muscle myoblast, and human diabetic skeletal muscle myoblast (DHSMM) cell lines were used as comparative in vitro models.

## 2. Results

**Figure 1:**
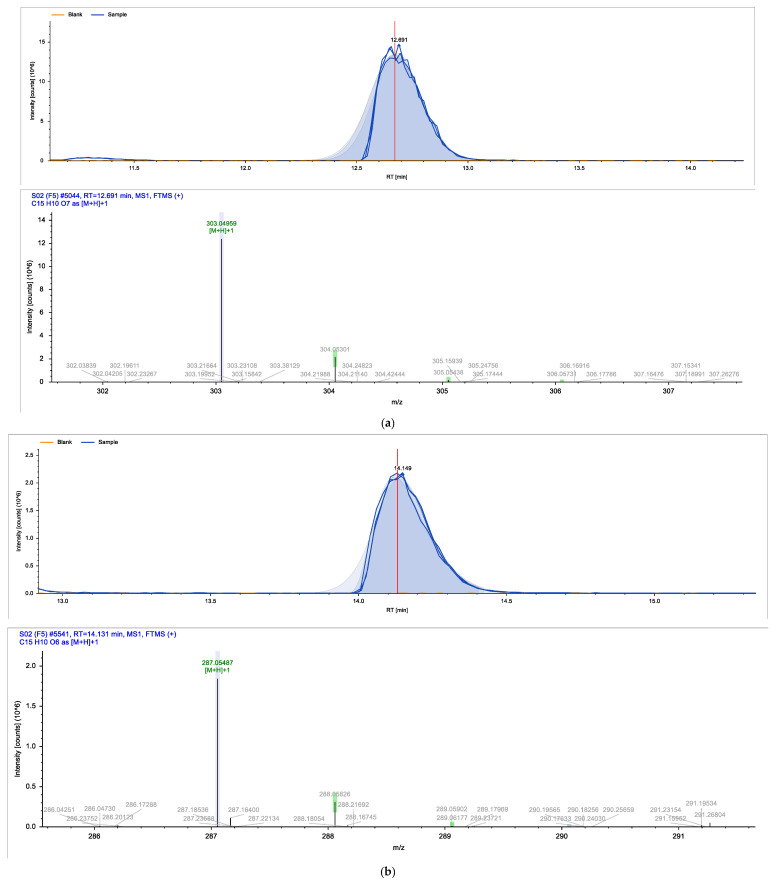

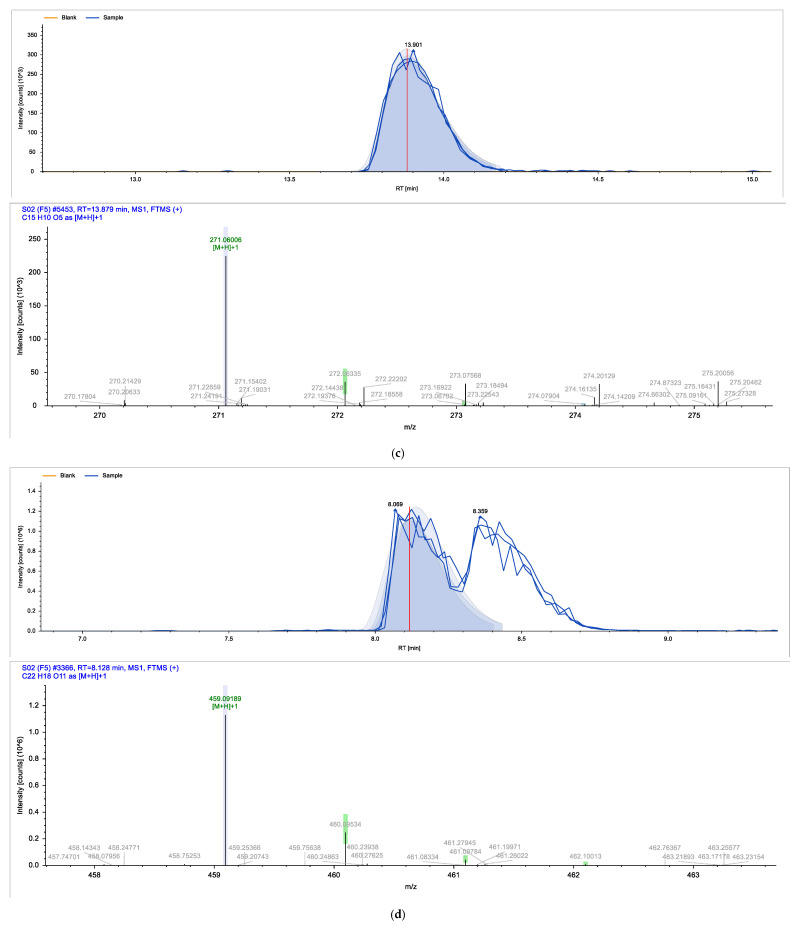

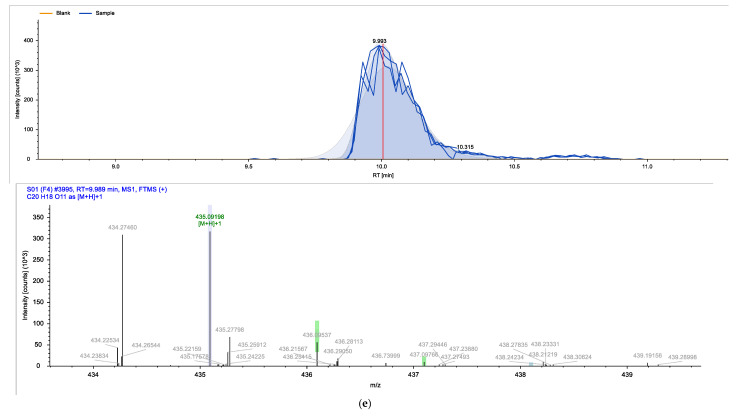
Shows the chromatogram of the five main chemical compounds in *K. pinnata* extract: (**a**) quercetin, (**b**) kaempferol, (**c**) apigenin, (**d**) epigallocatechin gallate, (**e**) avicularin.

Eighteen different phytocompounds were identified by ultraperformance liquid chromatography fusion orbitrap mass spectrometry (UPLC-OT-FTMS) in an aqueous *K. pinnata* extract (Table 2). The results showed that *K. pinnata* contained a wide variety of organic acids, flavonoids, and triterpenoids. Utilizing the mz Cloud database, mzlogic, and Arita Lab 6549 flavanoid structure database, five main phytocompounds were identified in the *K. pinnata* extract (Figure 1). The main constituents of *K. pinnata* detected were quercetin, kaempferol, apigenin, ECGC, and avicularin (Table 3). The chemicals reported from the UPLC-OT-FTMS data set were observed to be similar in chemical structure. The varying difference between the chemicals found in *K. pinnata* were the -OH functional groups, with the differences being where the hydroxyl group was placed. It was either shifted from the phenol functional group or displaced altogether. Regardless of the place of the hydroxyl functional group, the compounds were of the same chemical class called polyphenols, more specifically, flavonoids. Out of the five main phytochemicals identified, three of the compounds were flavanols, one a flavone, and another a catechin (Table 3). All compounds detected by UPLC-OT-FTMS are bioactive molecules that are known to display antioxidant activity.

Table 4 shows superoxide dismutase activity in treated and non-treated HSMM, stress-induced, and DHSMM cells. Superoxide dismutase activity in HSMM cells was not significantly altered among the untreated or two of the combinatorial treatment groups (150 μM metformin and 50 μg/mL of *K. pinnata*; 50 μM metformin and 150 μg/mL of *K. pinnata*). Other treatments showed a significant increase in SOD activity compared to the untreated control group. However, the group treated with metformin only was more effective in promoting SOD activity in HSMM cells. In H_2_O_2_-stress-induced HSMM cells, the combinatorial preparation containing 100 μM of metformin and 100 μg/mL of *K. pinnata* showed a significant increase in superoxide dismutase activity compared to all other treatments. In human diabetic skeletal muscle cells, a metformin-only treatment significantly increased the superoxide dismutase activity compared to all other treatments. Among the cell types, metformin-only treatment significantly lowered SOD activity in stress-induced HSMM and DHSMM compared to HSMM cells. Still, no significant changes were noted with the combination treatments except for the observed significant decrease in SOD activity in the combinatorial preparation containing 100 μM of metformin and 100 μg/mL of *K. pinnata* in the DHSMM compared to the stress-induced HSMM cells.

Table 5 exhibits the CAT activities in HSMM cells, the H_2_O_2_-stress-induced HSMM cell line, and DHSMM cells. Catalase activity was significantly elevated in HSMM cells when treated with 400 μg/mL of *K. pinnata*. Human skeletal muscle cells treated with metformin alone or a combination of 200 µM metformin and 25 µg/mL of *K. pinnata* did not significantly increase catalase activity compared to non-treated HSMM cells. However, all other combinations of metformin and *K. pinnata* were found to have significantly higher catalase activity compared to the control.

In H_2_O_2_-stress-induced human skeletal muscle myoblasts, 400 μg/mL *K. pinnata* significantly increased catalase activity compared to stress-induced myoblasts that received no treatment. A preparation of 50 μM of metformin combined with 150 μg/mL of *K. pinnata* was the only combinatorial preparation that did not significantly increase catalase activity compared to all other treatments.

Similarly, a *K. pinnata*-only treatment significantly increased catalase activity in the human diabetic skeletal muscle myoblast cell line compared to the control group. Combinatorial treatments containing metformin were found to produce significantly lower levels of catalase activity compared to the *K. pinnata*-only treatment. Non-treated diabetic myoblasts demonstrated the lowest catalase activity than those that were treated with the experimental preparations.

Overall, treatment with 400 μg/mL of *K. pinnata* increased catalase activity compared to all other treatment groups. All experimental treatments offered significant increases in catalase activity over the control.

Among the cell types there was a significant difference among cell types in the control group. The stress-induced HSMM showed the least activity of catalase, followed by DHSMM. A similar pattern was noted among the cell types treated with metformin-only treatment or *K. pinnata*-only treatment, or the combination treatment options except for the combinatorial preparation treatments containing 200 μM of metformin and 25 μg/mL of *K. pinnata* and the 150 μM of metformin and 50 μg/mL of *K. pinnata*. Catalase activity in the stress-induced HSMM was significantly lower compared to the HSMM and DHSMM cells.

Diabetic, non-diabetic, and stress-induced HSMM cells treated with combined metformin and *K. pinnata* preparations (50 μM metformin; 150 μg/mL *K. pinnata*) had significantly higher concentrations of reduced glutathione than any other treatment option (Table 6). The results from this study indicate that a combined preparation of 50 μM metformin with 150 μg/mL of *K. pinnata* significantly increases reduced glutathione concentrations in human skeletal myoblasts. Reduced glutathione content was significantly altered among the cell types in all the treatment options and the control.

According to Table 7, it was observed that all treatment options significantly decreased MDA concentrations in all human skeletal muscle cell lines. The most effective treatment in decreasing MDA concentrations was 400 μg/mL of *K. pinnata*. Among the cell types, the MDA level in control untreated cells was significantly higher in the stress-induced HSMM and DHSMM cells when compared to the HSMM cells. A similar pattern was noted in the combinatorial preparation containing 100 μM of metformin and 100 μg/mL of *K. pinnata.* The *K. pinnata*-only treatment showed a significant difference in MDA levels among the cell type with the least amount of MDA recorded in the DHSMM cells. Metformin-only treatment showed a significant decrease in MDA levels in DHSMM cells compared to the other cell types. The combinatorial preparation treatments containing 200 μM of metformin and 25 μg/mL of *K. pinnata* did not significantly alter the MDA levels among the cell types. Varied significant changes were noted in the other combinatorial preparation treatment options among the cell types.

## 3. Discussion

Currently, many in vitro and in vivo studies have focused on the role of phytochemicals in the activation of nuclear factor erythroid 2-related factor 2 (Nrf2), which leads to increased antioxidant capacity with a subsequent prevention of β-cell death. With the increase in the prevalence of type II diabetes and the emergence of alternative medicinal practices, it is vital to develop a combined multi-approach treatment option that is safe and effective in treating the impacts of oxidative stress in patients with diabetes.

In this study, we identified bioactive phytomolecules using UPLC-OT-FTMS in an aqueous *K. pinnata* extract that was locally grown in Corpus Christi, South Texas, USA, in the Gulf Coast region. The five main phytochemicals identified were quercetin, kaempferol, apigenin, ECGC, and avicularin. Observed antioxidant benefits in this study may be ascribed to the combined action of the bioflavonoids present in *K. pinnata*. Quercetin, a natural flavonoid detected in *K. pinnata*, has shown therapeutic potential against type II diabetes by acting as an anti-inflammatory and antioxidant. Quercetin has also exhibited similar effects as metformin [17,18]. By participating in and regulating signaling pathways, quercetin could protect cells from reactive oxygen species (ROS) and improve hyperglycemia by stimulating the production and release of insulin [18]. Epigallocatechin gallate (ECGC) has also been reported to regulate blood sugar and provide antioxidant benefits [19]. Researchers have shown that blood sugar levels were significantly reduced in mice given ECGC compared to mice without ECGC [20]. Another study showed that ECGC also decreased insulin resistance in human subjects given ECGC extracts and benefited those with type II diabetes [19,21]. Another bioactive constituent in *K. pinnata* is avicularin, which has been shown to reduce oxidative stress markers by modulating signaling pathways that are directly involved in cellular function and survival, such as the phosphoinositide 3-kinase (PI3K), Akt/PKB, and MAPK pathways [22]. Lee et al. demonstrated that avicularin possesses OH radical scavenging capacities [23]. Oxidative damage can be reduced and kept to a minimum by reducing hydroxyl radicals, which may, in turn, allow cell growth due to reduced inflammation caused by stress [24]. Although we did not investigate the effect of avicularin alone in our previous study that reported an increase in inflammatory markers, it may be due to the combined phytochemicals in the *K. pinnata* extract [14]. Like many of the other polyphenols previously mentioned, avicularin may offer help to type II diabetic patients. Studies have shown that avicularin can decrease the progression of type II diabetes by reducing levels of fasting blood glucose, triglycerides, and low-density lipoprotein cholesterol, improving β-cell function, and suppressing hepatic steatosis [24,25]. Kaempferol, another flavonoid found in *K. pinnata*, was shown to regulate concentrations of glucose within cells [26]. The results of this study match those observed in another research paper where kaempferol was detected in leaf juices from *K. pinnata* and displayed antioxidant properties [27]. Kaempferol significantly reduces malondialdehyde levels and supports antioxidant activity [27,28,29]. These findings in combination with the aforementioned studies reveal that *K. pinnata* possesses significant oxidative radical scavenging activities. The other component, apigenin, may also likely be a bioactive ingredient of *K. pinnata*. Apigenin, kaempferol, and resveratrol have been reported to reduce the expression and secretion of *TNF-α*. However, apigenin decreases the expression and secretion of *IL-10*, while kaempferol and resveratrol increase the secretion of IL-10 [30].

It is known that hydrogen peroxide is a relatively weak oxidizing agent but, at high levels, becomes cytotoxic [31]. However, hydrogen peroxide is an important regulator of cellular redox status and signaling pathways. When produced in excess, it can trigger oxidative damage, which can only be counteracted by antioxidant systems [32]. Oxidative stress plays an important role in the development of diabetic vascular complications, particularly type II diabetes [33,34]. Evidence has accumulated that ROS affect human skeletal muscle cells by influencing cellular processes that lead to increased expression of antioxidants [35,36]. Skeletal muscle tissue is persistently exposed to pro-oxidization because of its high oxygen consumption and metabolic rate [37]. A skeletal muscle environment with high ROS levels and reduced antioxidant defense is linked to oxidative stress that impairs physiological functions.

Antioxidants can neutralize free radicals produced by ROS by accepting unpaired electrons and inhibiting the oxidation of other cellular molecules that are vital to cellular survival. Depending on the oxygen consumption rate, cells constitutively express different levels of antioxidants, including mitochondrial antioxidant manganese superoxide dismutase (Mn-SOD, SOD2), cytosolic copper-zinc superoxide dismutase (Cu, Zn-SOD, SOD1), glutathione peroxidase (GPX) and catalase (CAT), and the non-enzymatic antioxidant glutathione (GSH) [35]. The non-enzymatic antioxidants, such as glutathione (GSH), maintain the cellular redox status, and the enzymatic antioxidants, such as superoxide dismutase and catalase, among others, constitute the defense system [38] https://www.ncbi.nlm.nih.gov/pmc/articles/PMC8400669/—B56-molecules-26-05041 (accessed on 14 February 2023).

In this study, a combinatorial preparation of 100 μM of metformin and 100 μg/mL of *K. pinnata* was most effective at increasing SOD activity in H_2_O_2_-stress-induced human skeletal muscle myoblasts and promoted cell viability after 24 h of treatment. However, the metformin-only treatment was most effective at increasing SOD activity in HSMM and DHSMM cell lines that were not H_2_O_2_-stress-induced, but was less effective at promoting cell viability after 72 h. This study reinforces an earlier report showing that metformin treatment significantly increases superoxide dismutase activity in a diabetic disease state [39]. However, significant increases in catalase activity for all cell lines occurred when cells were treated with 400 μg/mL of *K. pinnata*. This study suggests that 400 μg/mL of *K. pinnata* may reduce oxidative damage by increasing catalase activity in human skeletal muscle cells compared to other treatment groups and may be beneficial in reducing oxidative stress in type II diabetes [40]. The increase in catalase activity in the *K. pinnata*-only treatment may be associated with the combined action of the bioactive compounds in the extract, as similarly reported in another study [10]. It should be noted, however, that catalase has a relatively low affinity for H_2_O_2_ when compared with other peroxidases [41], which may account for the earlier reported [14] decrease in the HSMM H_2_O_2-_stress-induced cell viability after 72 h of treatment. The rate of H_2_O_2_ detoxification increases with the intracellular concentration of catalase, preventing H_2_O_2_ accumulation and subsequent oxidative injury under conditions where other peroxidases are saturated [42,43]. Our data show that H_2_O_2_ detoxification by catalase is effective at promoting cell viability in H_2_O_2_-stress-induced HSMM after 24 h of treatment and less effective after 72 h. Although catalase activity was highest in H_2_O_2_-stress-induced HSMM treated with *K. pinnata* only, it did not support the viability of cells after 72 h, even though the MDA concentration was lowest among the treatment groups. The catalase content of skeletal muscle tissue has been identified to be relatively low, with only 1.4% of that found in the liver [41].

Combinatorial treatment of 50 μM metformin and 150 μg/mL *K. pinnata* significantly increased reduced glutathione concentrations in diabetic, non-diabetic, and H_2_O_2_-stress-induced human skeletal muscle myoblasts. Although it suggests this combinatorial preparation is more effective at increasing reduced glutathione levels and protecting against oxidative stress, it was less effective at promoting cell viability in DHSMM cells compared to 100 μM metformin and 100 μg/mL *K. pinnata*; 150 μM metformin and 50 μg/mL *K. pinnata*; 200 μM metformin and 25 μg/mL *K. pinnata;* and metformin-only treatment groups after 72 h. Nuclear factor erythroid 2-related factor 2 regulates the antioxidant enzyme system that prevents oxidative damage. However, the optimal intracellular level of glutathione involves synthesis, consumption, and regeneration that encompasses a network of antioxidant enzymes regulated by Nrf2 [44]. Hence, the combinatorial treatment of 50 μM metformin and 150 μg/mL *K. pinnata* may be more effective at activating Nrf2 that favors the regeneration of GSH. Reduced glutathione elevation has also been reported to improve sugar metabolism in patients with type II diabetes [45]. Researchers from the University of Rome found that increasing concentrations of reduced glutathione in type II diabetic cells significantly increased glucose uptake and sensitivity to insulin [46]. In addition, impaired fibroblast growth was prevented by increasing reduced glutathione concentrations [46]. This suggests that oxidative stress is an important contributor to cellular dysfunction in a type II diabetic disease state and that GSH supplementation can protect the body against oxidative stress and improve cell metabolism [47].

All treatment options resulted in a reduction in lipid peroxidation in DHSMM cells, which may subsequently prevent oxidative damage from occurring in the cell. A *K. pinnata*-only treatment significantly reduced MDA concentrations for all cell lines and outperformed every other treatment option in reductions in lipid peroxidation. Hence, *K. pinnata* alone is better suited for protecting human skeletal muscle myoblasts from oxidative damage caused by lipid peroxidation. In another study, concentrations of *K. pinnata* extract at 125 μg/mL produced an antilipid peroxidation effect on in vitro cells where antioxidation increased as *K. pinnata* concentrations increased [48].

Among the cell type, the observed increase in cell viability may not be solely linked to the countering effects of the antioxidant activity of *K. pinnata*, as we noted that even though the *K. pinnata*-only treatment showed the least amount of MDA levels in the DHSMM cells it did not correlate with cell viability in the DHSMM cells after 72 h of treatment. Similarly, the other treatment options’ antioxidant activity did not correlate with the observed cell viability among the cell types after 72 h of treatment.

## 4. Materials and Methods

### 4.1. Preparation of K. pinnata Extract

Ten broad leaves from *K. pinnata* were selected from the middle of the plant in the fall season and weighed for extract preparation [49]. Ten leaves of *K. pinnata* (average weight of 4.30 g) were ground up, mixed with 100 mL of distilled water, and stirred at 71 °C for 12 h [50]. The solution was then filtered by gravity filtration so that any insoluble materials were removed, and the supernatant was freeze-dried. The resulting extract was reconstituted with distilled water for the diluted concentrations. In addition, for subsequent cell culture experiments, the *K. pinnata* leaf extract was then dissolved in dimethyl sulphoxide (DMSO) (Corning Cellgro, Manassas, VA, USA) and then passed through a 0.20 µm filter. Working concentrations of the plant extract at 0, 25, 50, 100, 150, 200, 300, and 400 μg/mL were prepared by diluting the stock solution with DMSO. The concentration of DMSO solutions never exceeded 1% of the total volume [51,52,53].

### 4.2. Determination of Chemical Composition of K. pinnata

A 1 mL aliquot of the *Kalanchoe pinnata* extract solution was analyzed by a Vanquish Ultra Pressure Liquid Chromatography–Orbitrap Fusion Tribrid Mass Spectrometer (UPLC-OT-FTMS, Tokyo, Japan). The analytes were separated on the 1.7 μm ACQUITY UPLC BEH C_18_ reversed-phase column by Waters (130 Å, 1.7 μm, 2.1 mm × 150 mm). Eluent A, Milli-Q with 0.1% (*v*/*v*) formic acid, and eluent B, acetonitrile with 0.1% (*v*/*v*) formic acid, were mixed with curve 5 to a flow rate of 0.200 mL/min. The total run lasted 31 min with 7 min re-equilibration and the following gradient: 0–2 min hold at 5% B, ramp to 65% B for 18 min, then ramp to 100% B for 1 min and hold at 100% B for 3 min. The H-ESI setting was 3500 V for the positive spray voltage with the ion transfer tube temperature at 300 °C and vaporization temperate at 225 °C. The three gases on the H-ESI were 35 for sheath gas, 7 for aux gas, and 0 for sweep gas. The Orbitrap was run at a 120,000 (FWHM at *m*/*z* 200) resolution and mass range 85–700 *m*/*z* with an RF lens at 40%. Following the full scan, two MS^2^ were scanned with the ion trap via two filters, Dynamic Exclusion (*n* = 3 for 60 s) and intensity threshold (min = 1000, max = 1.0e20). Both MS^2^ scans were isolated with the Quadrupole (0.7 *m*/*z*), but one fragmentation scan was generated through CID with assisted energy collision, and the other fragmentation scan was generated through HCD with stepped energy collision. MS^2^ scan with CID had an automatic gain control (AGC) set at 3.0e4 and a maximum injection time of 50 ms, and the MS^2^ scan with HCD had an AGC of 1.0e4 and a maximum injection time of 50 ms. Compound Discoverer 3.2 (Thermo Scientific, Tokyo, Japan) was used to identify the compounds in the *K. pinnata* aqueous extract. The adaptive curve was used with a 2 min maximum shift and 5 ppm mass tolerance to align the retention times of chromatography spectra. For compound identification, the following four criteria were met: (1) a signal-to-noise threshold of 3; (2) a minimum of 8 scans per peak; (3) a minimum of one isotope; and (4) a peak intensity of 50,000. The detected compounds were then compared using the mzCloud database (Thermo Fisher). For further verification, the collected MS^2^ spectral data were processed by Thermo ScientificTM Mass FrontierTM 8.0 software to ensure compounds included the basic flavonoid structure and thus belonged to the flavonoid class. These detected flavonoid-related compounds were then further annotated using an Arita Lab 6549 flavonoid structure database (Professor Masanori Arita from National Institute of Genetics, Japan) and structure ranking tools within Thermo Scientific Compound Discoverer 3.2 software.

### 4.3. Preparation of Metformin Solutions

Metformin tablets (500 mg) (Bristol-Meyers Squibb Company, New York, NY, USA) were crushed with a pestle and mortar and suspended in distilled water to create 0, 10, 30, 50, 100, 150, 200 μM, and 5 mM solutions. A 5 mM metformin-concentration-only treatment option was examined as it is an effective dose for cultured diabetic cells [54,55,56]. Micromolar concentrations of metformin have been reported to potentially have protective properties against cell death and senescence [57]. According to He et al., low metformin concentrations (<50 μM) are achieved in the portal vein as well as in tissue circulation [58]. He et al. also suggested that metformin concentrations greater than 80 μM are unachievable in the portal vein and are therefore clinically irrelevant or even toxic [58]. Therefore, a range of 0–200 μM for the combination preparations and one 5 mM concentration of metformin were investigated.

### 4.4. Combinatorial K. pinnata and Metformin Preparations

The concentrations for *K. pinnata* in combination with metformin ranged from 0–400 μg/mL, due to similar ranges used in other studies [15,59,60]. A relatively high dose of 400 μg/mL of *K. pinnata* only was chosen as a treatment option since higher concentrations of phytochemicals are present in larger concentrations of crude *K. pinnata* extracts [61]. The combinatorial ratios vary between the 5.0 mM metformin-only and 400 μg/mL *K. pinnata*-only preparations to create differing treatment options. HSMM and DHSMM cells were treated with the preparations for 48 h, after which cell pellets were harvested for the assays of lipid peroxidation and reduced glutathione levels, along with superoxide dismutase and catalase activities.

### 4.5. Preparation of Cultured Human Skeletal Muscle Cells

Human skeletal muscle myoblast (HSMM) and human diabetic muscle myoblast (DHSMM) cells (Lonza, Rockville, MD, USA) were cultured at a concentration of 10^5^ cells/mL in skeletal muscle growth media-2 (SkGM™-2 Medium) containing human Epidermal Growth Factor (hEGF), Dexamethasone, L-glutamine, Fetal Bovine Serum (FBS), Gentamicin/Amphotericin-B (GA), 50 U/mL of penicillin, and 50 mg/mL of streptomycin. The cell suspension was incubated at 37 °C in a humidified atmosphere containing 5% CO_2_. When cell growth reached 10^6^ cells/mL, cells were harvested according to the manufacturer’s instructions (Lonza, MD, USA) for assays.

### 4.6. Induction of Oxidative Stress on HSMM Cell Culture

Three separate sets of cultured cells (one control, one under oxidative stress, and one diabetic) were used to determine how a combined preparation of *K. pinnata* and metformin will affect cultured cells under the duress of oxidative stress. To induce oxidative stress in the cultured cells, a concentration of 0.1 M of H_2_O_2_ was added to a fresh cell culture medium. The final working concentration of hydrogen peroxide in the cell culture was 50 μM. Afterwards, the cell cultures were suspended in the prepared medium containing 50 μmol of H_2_O_2_ and the cells were adjusted to a concentration of 10^6^ cells/mL then treated with the preparations for 48 h. Following the treatment, cell pellets were harvested for the assays of lipid peroxidation and reduced glutathione levels, along with superoxide dismutase and catalase activities.

### 4.7. Determination of Cell Concentration

The number of viable cells was assessed by a 0.4% trypan blue solution. A volume of 100 μL of 0.4% trypan blue solution was transferred to a 0.5 mL microcentrifuge tube followed by adding 100 μL of cell suspension and vortexed thoroughly. The dilution factor used throughout this study was 2. Ten microliters of the cellular solution were pipetted into a hemocytometer chamber and covered with a glass coverslip. The number of cells in the one-millimeter center square and four one-millimeter corner squares were counted. A factor of 10^4^ was used in cell counting to take into account that each square has a volume of 0.0001 mL (1 mm × 1 mm × 0.1 mm = 0.1 mm^3^). Non-viable cells were stained blue while viable cells remained transparent. The total number of cells per unit volume was determined by the following formula:

Cells/mL = average viable cells counted/number of squares counted × dilution factor × 10^4^

### 4.8. Measurement of Superoxide Dismutase Activity

Cell pellets (10^6^ cells/mL) were lysed by sonication in a cold 20 mM Hepes buffer (pH = 7.2) containing 1 mM EGTA, 210 mM mannitol, and 70 mM sucrose. The lysate was then centrifuged for 5 min. Cellular supernatant was used for the measurement of superoxide dismutase activity. A one-milliliter reaction mixture contained 500 μL of 0.1 M sodium phosphate buffer, 32 μL of 3.3 mM ethylenediaminetetraacetic acid (EDTA), 60 μL of 8.1 mM pyrogallol, and an appropriate amount of cellular supernatant that contained 11.51 mg/dL of protein. The enzymatic activity of superoxide dismutase was determined by measuring a change in absorbance at 420 nm against a blank for 2 min that contained all the ingredients except the cellular supernatant. One unit of the enzyme was defined as the amount of enzyme that caused a half-maximal inhibition of pyrogallol autoxidation [62].

### 4.9. Measurement of Catalase Activity

Cells were collected by centrifugation at 2000× *g* for 10 min at 4 °C. The cell pellet was sonicated on ice in 1–2 mL of cold buffer (50 mM potassium phosphate, pH 7.0 containing 1 mM EDTA) and then centrifuged at 10,000× *g* for 15 min at 4 °C. The supernatant was removed for the catalase assay. The catalase assay was conducted according to the method of Sinha [63]. Briefly, 200 μL of extract sample was added with 400 μL 0.1 M phosphate buffer (pH = 7.5) and 400 μL of 0.2 M H_2_O_2_ to create a 1 mL sample solution. Two milliliters of 5% potassium dichromate and glacial acetic acid (1:3 *v*/*v*) was added to the sample. The mixture was then heated in a water bath for 10 min and allowed to cool. The absorbance was read at 570 nm against a reagent blank using a spectrophotometric plate reader.

### 4.10. Determination of Reduced Glutathione

The reduced GSH levels were measured by following Elman’s method [64]. The harvested pellets were lysed in a hypotonic solution for 45 min at 37 °C and then processed for the assay [65]. One hundred microliters of the lysate was mixed with 10% trichloroacetic acid (TCA) and centrifuged at 2000× *g* for 15 min. One milliliter of supernatant was treated with Ellman’s reagent (19.8 mg of 5,5′-dithiobis-2-nitrobenzoic acid (DTNB) in 100 mL of 0.2 phosphate buffer (pH 8)). The absorbance was read at 412 nm.

### 4.11. Determination of Malondialdehyde (MDA)

This study utilized the thiobarbituric acid reacting substances test (TBARS) method for investigating lipid peroxidation in cells after the treatment of a combined *K. pinnata* and metformin preparation. Cells were lysed in ice-cold physiological saline, followed by centrifugation at 28,000× *g* for 5 min at 4 °C [66]. The cellular supernatant was used for the measurement of MDA [66]. The supernatant was mixed with thiobarbituric acid (TBA). Samples and MDA standards (16.7, 8.35, 4.18, 2.09, 1.04, 0.52, 0.26, and 0 μM) were acid-treated with 10% (*w*/*v*) trichloroacetic acid (TCA) [67]. A volume of 150 μL of MDA standards and cellular supernatant was added to the wells of a 96-well plate. Afterward, 75 μL of TBA was added to each well. The absorbance of each well was pre-read using a microplate reader set to 532 nm. Once the pre-read absorbance data were collected, the well plate was covered with a plate cover and incubated for 3 h at 50 °C. Absorbance was collected again after incubation using a microplate reader set to 532 nm. The pre-read absorbance was subtracted from the final absorbance reading to correct for the sample’s contribution to the final absorption at 532 nm. A standard curve was constructed from the standards and the concentrations of MDA within the samples were extrapolated from the standard curve. The total protein in the cellular supernatant was determined using a Stanbio kit [68].

### 4.12. Statistical Analysis

All data were obtained from three separate experiments, and each experiment included three controls (metformin only, *K. pinnata* extract only, and untreated) and combined *K. pinnata* and metformin preparations performed in triplicate. Untreated cells were those without any treatment and were used as a control. The data are presented as the mean ± standard error of the mean. The results among different concentrations were evaluated by two-way ANOVA (*p* < 0.05). Post hoc analysis was performed using Tukey’s multiple comparison (*p* < 0.05) to test for a significant difference among the means.

## 5. Conclusions

This study showed that a *K. pinnata*-only treatment effectively increased CAT activity and significantly lowered MDA concentrations in diabetic and non-diabetic human skeletal muscle cells. This benefit may be due to the bioactive compounds with conjugated rings identified in *K. pinnata*. A combinatorial treatment promoted higher concentrations of reduced glutathione that may prevent oxidative damage in HSMM and DHSMM cells. A metformin-only treatment was most effective at significantly increasing SOD activity in both HSMM and DHSMM cells. These treatment options show promise for combating oxidative-stress-mediated pathophysiological complications caused by type II diabetes. The data presented in this study suggest that combinatorial treatment could be a potential source of combined natural antioxidants that may act synergistically with metformin and accrue greater effectiveness in treating radical-related diseases. However, the treatment options did not promote cell viability after 72 h in H_2_O_2_-stress-induced HSMM cells. Overall, the antioxidant activity of the treatment options appears to correlate best with cell viability after 24 h of treatment versus 72 h. Treatment of *K. pinnata*-only was more effective at lowering oxidative stress damage associated with MDA levels in the DHSMM cells after 72 h of treatment. Further studies are needed to assess the antioxidant activity after 24 h of treatment and the bioavailability of the identified compounds and establish a more precise/appropriate dosing regimen. Moreover, an in-depth further molecular study of the combination treatment on Nrf2 activation after 24 h of treatment is required.

## Figures and Tables

**Table 1:** Cell viability represented as a percentage of the control after 72 h of treatment. Data are shown as mean ± S.E.M. Significant differences are indicated by different letters (uppercase letters within row; lowercase letter within column for each treatment option) according to Tukey post hoc test (*p* < 0.05): control (no treatment); human skeletal muscle myoblast (HSMM); diabetic human skeletal muscle myoblast (DHSMM).

Treatment		Day 1 (24 h)			Day 3 (72 h)		
Metformin (µm)	*K. pinnata* (µg/mL)	HSMM Cell Viability (% of Control)	DHSMM Cell Viability (% of Control)	Stress-Induced Cell Viability (% of Control)	HSMM Cell Viability (% of Control)	DHSMM Cell Viability (% of Control)	Stress-Induced Cell Viability (% of Control)
5000	0	98.6 ± 0.907 ^Aa^	99.27 ± 2.78 ^Aa^	97.90 ± 0.004 ^Aa^	86.92 ± 0.242 ^Aa^	103.90 ± 0.007 ^Ba^	75.22 ± 0.081 ^Ca^
200	25	97.49 ± 0.907 ^Aa^	98.9 ± 2.78 ^Aa^	100.95 ± 0.068 ^Ab^	104.93 ± 0.015 ^Ac^	104.95 ± 0.006 ^Aa^	63.47 ± 0.048 ^Bc^
150	50	98.05 ± 0.907 ^Aa^	94.9 ± 2.78 ^Aa^	100.66 ± 0.03 ^Ab^	88.54 ± 0.104 ^Aa^	104.66 ± 0.006 ^Ba^	63.25 ± 0.078 ^Cc^
100	100	97.02 ± 0.907 ^Aa^	94.9 ± 2.78 ^Aa^	101.02 ± 0.15 ^Ab^	100.13 ± 0.143 ^Ac^	105.02 ± 0.002 ^Ba^	58.02 ± 0.067 ^Cc^
50	150	95.76 ± 0.907 ^Aa^	96.5 ± 2.78 ^Aa^	96.48 ± 0.016 ^Ac^	99.34 ± 0.156 ^Ac^	101.48 ± 0.007 ^Bb^	48.91 ± 0.125 ^Cd^
30	200	96.39 ± 0.907 ^Aa^	94.7 ± 2.78 ^Aa^	95.10 ± 0.045 ^Ac^	98.10 ± 0.166 ^Ac^	102.10 ± 0.006 ^Bb^	44.65 ± 0.048 ^Cd^
10	300	94.12 ± 0.907 ^Aa^	80 ± 2.78 ^Ab^	95.67 ± 0.046 ^Ac^	82.25 ± 0.315 ^Ab^	102.67 ± 0.005 ^Bb^	40.19 ± 0.10 ^Cd^
0	400	90.7 ± 0.907 ^Ab^	80 ± 2.78 ^Ab^	99.82 ± 0.042 ^Aa^	83.41 ± 0.292 ^Ab^	101.82 ± 0.01 ^Bb^	20.81 ± 0.014 ^Cb^

**Table 2:** Phytochemical profiling of aqueous *K. pinnata* extract using UPLC-OT-FTMS.

Identified Compound	Retention Time (min)	Chemical Formula	Molecular Weight (g/mol)
(2R,3R)-Taxifolin	6.449	C_15_H_12_O_7_	304.25
Epigallocatechin gallate	8.128	C_22_H_18_O_11_	458.37
Luteolin-7-glucoside	9.817	C_21_H_20_O_11_	448.37
Scutellarin	9.822	C_21_H_18_O_12_	462.36
Myricetin	9.909	C_15_H_10_O_8_	318.24
Avicularin	9.989	C_20_H_18_O_11_	434.35
Hispidulin 7-glucuronide	10.698	C_22_H_20_O_12_	476.4
Genistin	10.71	C_21_H_20_O_10_	432.37
Baicalin	10.764	C_21_H_18_O_11_	446.36
Prunin	10.791	C_21_H_22_O_10_	434.4
Juglanin	10.805	C_20_H_18_O_10_	418.35
Tricin 5-glucoside	11.117	C_23_H_24_O_12_	492.4
Afzelin	11.289	C_21_H_20_O_10_	432.38
Eriodictyol	12.546	C_15_H_12_O_6_	288.25
Quercetin	12.691	C_15_H_10_O_7_	302.24
Apigenin	13.879	C_15_H_10_O_5_	270.05
Chalconaringenin	13.971	C_15_H_12_O_5_	272.26
Kaempferol	14.131	C_15_H_10_O_6_	286.23

**Table 3:** Main compounds identified with UPLC-OT-FTMS in *K. pinnata* extract along with their structure and classification.

Compound Name	Compound Structure	Chemical Class
Quercetin	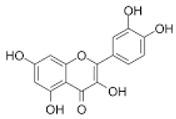	Flavonol
Kaempferol	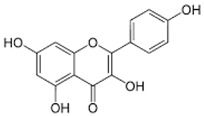	Flavonol
Apigenin	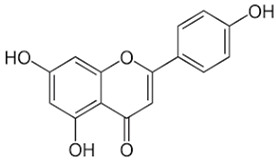	Flavone
Epigallocatechin gallate (EGCG)	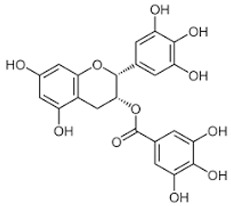	Catechin
Avicularin	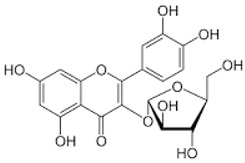	Flavonol

**Table 4:** Observed cellular superoxide dismutase (SOD) activity in cells after 72 h of treatment. Data are presented as mean ± standard error of the mean. Significant differences are indicated by different letters (uppercase letters within row; lowercase letter within column for each treatment option) according to Tukey post hoc test (*p* < 0.05): control (no treatment); human skeletal muscle myoblast (HSMM); diabetic human skeletal muscle myoblast (DHSMM).

Metformin (μM)	*K. pinnata* (μg/mL)	HSMM SOD Activity (U/mg of Protein/min)	Stress-Induced SOD Activity (U/mg of Protein/min)	DHSMM SOD Activity (U/mg of Protein/min)
Control (0)	Control (0)	60.48 ± 4.68 ^Aa^	57.68 ± 7.63 ^Aa^	40.35 ± 10.95 ^Aa^
5000	0	137.81 ±15.80 ^Ab^	77.89 ± 12.31 ^Bb^	65.91 ± 11.48 ^Bb^
0	400	71.90 ± 12.94 ^Ac^	83.89 ± 17.68 ^Ab^	59.92 ± 14.63 ^Ac^
200	25	68.91 ± 3.34 ^Ac^	59.92 ± 17.30 ^Aa^	44.94 ± 14.31 ^Aa^
150	50	59.92 ± 6.40 ^Aa^	56.92 ± 11.83 ^Aa^	59.92 ± 12.06 ^Ac^
100	100	86.88 ± 10.91 ^ABd^	107.85 ± 24.48 ^Ac^	59.92 ± 14.35 ^Bc^
50	150	62.91 ± 5.57 ^Aa^	56.92 ± 8.04 ^Aa^	56.92 ± 13.16 ^Ac^
30	200	68.91 ± 9.12 ^Ac^	59.92 ± 13.68 ^Aa^	47.93 ± 6.61 ^Aa^
10	300	71.9 ± 6.97 ^Ac^	59.92 ± 12.06 ^Aa^	56.92 ± 10.20 ^Ac^

**Table 5:** Observed cellular catalase (CAT) activity in cells after 72 h of treatment. Data are presented as mean ± standard error of the mean. Significant differences are indicated by different letters (uppercase letters within row; lowercase letter within column for each treatment option) according to Tukey post hoc test (*p* < 0.05): control (no treatment); human skeletal muscle myoblast (HSMM); diabetic human skeletal muscle myoblast (DHSMM).

Metformin (μM)	*K. pinnata* (μg/mL)	HSMM CAT Activity (U/mg of Protein/min)	Stress-Induced CAT Activity (U/mg of Protein/min)	DHSMM CAT Activity (U/mg of Protein/min)
Control (0)	Control (0)	50.32 ± 3.36 ^Aa^	10.15 ± 4.12 ^Ba^	30.26 ± 3.36 ^Ca^
5000	0	53.86 ±3.37 ^Aa^	22.13 ± 0.79 ^Bb^	38.06 ± 6.56 ^Cb^
0	400	86.88 ± 2.16 ^Ab^	50.83 ± 9.23 ^Bc^	66.27 ± 0.12 ^Cc^
200	25	55.22 ± 3.49 ^Aa^	17.74 ± 2.35 ^Bb^	45.65 ± 0.22 ^Ad^
150	50	64.72 ± 0.30 ^Ac^	44.35 ± 9.22 ^Bd^	62.5 ± 3.58 ^Ae^
100	100	79.48 ± 7.61 ^Ab^	30.83 ± 0.65 ^Be^	58.36 ± 0.16 ^Ce^
50	150	64.48 ± 4.41 ^Ac^	13.11 ± 2.43 ^Ba^	41.94 ± 0.97 ^Cd^
30	200	64.60 ± 1.67 ^Ac^	42.93 ± 5.55 ^Bd^	53.24 ± 1.36 ^ABf^
10	300	80.34 ± 1.17 ^Ab^	22.93 ± 2.28 ^Bb^	44.91 ± 1.68 ^Cd^

**Table 6:** Reduced glutathione content in cells after 72 h of treatment. Data are presented as mean ± standard error of the mean. Significant differences are indicated by different letters (uppercase letters within row; lowercase letter within column for each treatment option) according to Tukey post hoc test (*p* < 0.05). Control (No treatment); Human skeletal muscle myoblast (HSMM); Diabetic human skeletal muscle myoblast (DHSMM).

Metformin (μM)	*K. pinnata* (μg/mL)	HSMM (GSH) (μM)	Stress-Induced (GSH) (μM)	DHSMM (GSH) (μM)
Control (0)	Control (0)	2.12 ± 0.04 ^Aa^	3.11 ± 0.03 ^Ba^	5.25 ± 0.03 ^Ca^
5000	0	13.33 ± 0 ^Ab^	10.00 ± 0.10 ^Bb^	13.89 ± 0.03 ^Cb^
0	400	2.22 ± 0.02 ^Aa^	3.89 ± 0.02 ^Ba^	21.67 ± 0 ^Cc^
200	25	6.11 ± 0.05 ^Ac^	5.00 ± 0.06 ^Bc^	11.67 ± 0 ^Cb^
150	50	6.66 ± 0.06 ^Ac^	10.00 ± 0.03 ^Bb^	15.00 ± 0.03 ^Cb^
100	100	17.22 ± 0.25 ^Ad^	9.44 ± 0.07 ^Bd^	11.11 ± 0.05 ^Cb^
50	150	17.77 ± 0.06 ^Ad^	22.22 ± 0.03 ^Be^	23.89 ± 0.07 ^Cc^
30	200	9.44 ± 0.02 ^Ae^	11.67 ± 0.03 ^Bf^	14.44 ± 0.03 ^Cb^
10	300	8.89 ± 0.02 ^Ae^	11.67 ± 0.05 ^Bf^	10.56 ± 0.07 ^Cb^

**Table 7:** Cellular MDA concentrations in cells after 72 h of treatment. Data are presented as mean ± standard error of the mean. Significant differences are indicated by different letters (uppercase letters within row; lowercase letter within column for each treatment option) according to Tukey post hoc test (*p* < 0.05). Control (No treatment); Human skeletal muscle myoblast (HSMM); Diabetic human skeletal muscle myoblast (DHSMM).

Metformin (μM)	*K. pinnata* (μg/mL)	HSMM (MDA) (µM)	Stress-Induced (MDA) (µM)	DHSMM (MDA) (µM)
Control (0)	Control (0)	52.35 ± 1.25 ^Aa^	62.32 ± 1.1 ^Ba^	63.45 ± 0.98 ^Ba^
5000	0	25.15 ±0.11 ^Ab^	23.63 ± 0.24 ^Ab^	21.42 ± 0.30 ^Bb^
0	400	21.51 ± 0.64 ^Ac^	18.26 ± 0.88 ^Bc^	15.71 ± 0.50 ^Ce^
200	25	22.63 ± 0.47 ^Ac^	21.21 ± 0.69 ^Ae^	21.55 ± 1.15 ^Ab^
150	50	21.55 ± 0.11 ^Ac^	20.39 ± 0.30 ^ABe^	19.35 ± 0.22 ^Bc^
100	100	24.32 ± 0.61 ^Ab^	22.07 ± 0.49 ^Bd^	20.73 ± 0.24 ^Bd^
50	150	22.42 ± 0.28 ^Ac^	20.77 ± 0.24 ^ABe^	19.26 ± 0.13 ^Bc^
30	200	22.25 ± 0.13 ^Ac^	20.47 ± 0.46 ^ABe^	18.87 ± 0.27 ^Bc^
10	300	21.38 ± 0.16 ^Ac^	19.91 ± 0.30 ^ABe^	18.31 ± 0.50 ^Bc^

## Data Availability

No new data were created or analyzed in this study. Data sharing is not applicable to this article.

## References

[1] National Institutes of Health (2017). Diabetes. https://medlineplus.gov/diabetes.html.

[2] Lehrke M., Marx N. (2017). Diabetes Mellitus and Heart Failure. Am. J. Med..

[3] Kawahito S., Kitahata H., Oshita S. (2009). Problems associated with glucose toxicity: Role of hyperglycemia-induced oxidative stress. World J. Gastroenterol..

[4] Sakai K., Matsumoto K., Nishikawa T., Suefuji M., Nakamaru K., Hirashima Y., Kawashima J., Kawashima T., Ichinose K., Brownlee M. (2003). Mitochondrial reactive oxygen species reduce insulin secretion by pancreatic beta-cells. Biochem. Biophys. Res. Commun..

[5] Matough F.A., Budin S.B., Hamid Z.A., Alwahaibi N., Mohamed J. (2012). The role of oxidative stress and antioxidants in diabetic complications. Sultan Qaboos Univ. Med. J..

[6] Pfeiffer A.F.H., Klein H.H. (2014). The treatment of type 2 diabetes. Dtsch. Arztebl. Int..

[7] Cawich S.O., Harnarayan P., Budhooram S., Bobb N.J., Islam S., Naraynsingh V. (2014). Wonder of Life (*Kalanchoe pinnata*) leaves to treat diabetic foot infections in Trinidad & Tobago: A case control study. Trop. Dr..

[8] Joseph B., Sridhar S., Sankarganesh J., Edwin B.T. (2011). Rare Medicinal Plant-*Kalanchoe pinnata*. Res. J. Microbiol..

[9] Pattewar S.V. (2012). *Kalanchoe pinnata*: Phytochemical and Pharmacological Profile. Int. J. Phytopharm..

[10] Uchegbu R.I., Ahuchaogu A.A., Amanze K.O., Ibe C.O. (2017). Chemical Constituents Analysis of the Leaves of Bryophyllum pinnatum by GC-MS. AASCIT J. Chem..

[11] Eruygur N., Ucar E., Ataş M., Ergul M., Ergul M., Sozmen F. (2019). Determination of biological activity of Tragopogon porrifolius and Polygonum cognatum consumed intensively by people in Sivas. Toxicol. Rep..

[12] El-Hamamsy M.H.R.I. (2005). Potential Antimycobacterial Agents Targeting Dihydrofolate Reductase.

[13] El-Hamamsy M.H.R.I., Smith A.W., Thompson A.S., Threadgill M.D. (2007). Structure-based design, synthesis and preliminary evaluation of selective inhibitors of dihydrofolate reductase from Mycobacterium tuberculosis. Bioorg. Med. Chem..

[14] Ramon P., Sparks J., Omoruyi F. (2021). Effect of Combined *K. pinnata* and Metformin Preparation on Inflammatory Cytokines in Normal and Diabetic Skeletal Muscle Cells. J. Med. Food.

[15] Agarwal H., Shanmugam V.K. (2019). Anti-inflammatory activity screening of *Kalanchoe pinnata* methanol extract and its validation using a computational simulation approach. Inform. Med. Unlocked.

[16] Hernández-Caballero M.E., Sierra-Ramírez J.A., Villalobos-Valencia R., Seseña-Méndez E. (2022). Potential of *Kalanchoe pinnata* as a Cancer Treatment Adjuvant and an Epigenetic Regulator. Molecules.

[17] Sajan M.P., Bandyopadhyay G., Miura A., Standaert M.L., Nimal S., Longnus S.L., Van Obberghen E., Hainault I., Foufelle F., Kahn R. (2010). AICAR and metformin, but not exercise, increase muscle glucose transport through AMPK-, ERK-, and PDK1-dependent activation of atypical PKC. Am. J. Physiol.-Endocrinol. Metab..

[18] Dhanya R. (2021). Quercetin for managing type 2 diabetes and its complications, an insight into multitarget therapy. Biomed. Pharmacother..

[19] A Complete Guide to EGCG. https://compoundingrxusa.com/blog/your-guide-to-egcg-epigallocatechin-3-gallate/.

[20] Forester S.C., Gu Y., Lambert J.D. (2012). Inhibition of starch digestion by the green tea polyphenol, (−)-epigallocatechin-3-gallate. Mol. Nutr. Food Res..

[21] Liu C.Y., Huang C.J., Huang L.H., Chen I.J., Chiu J.P., Hsu C.H. (2014). Effects of Green Tea Extract on Insulin Resistance and Glucagon-Like Peptide 1 in Patients with Type 2 Diabetes and Lipid Abnormalities: A Randomized, Double-Blinded, and Placebo-Controlled Trial. PLoS ONE.

[22] Williams R.J., Spencer J.P.E., Rice-Evans C. (2004). Flavonoids: Antioxidants or signalling molecules?. Free Radic. Biol. Med..

[23] Lee J.S., Lee A.Y., Quilantang N.G., Geraldino P.J.L., Cho E.J., Lee S. (2019). Anti-oxidant activity of avicularin and isovitexin from *Lespedeza cuneate*. J. Appl. Biol. Chem..

[24] Wang Y., Liu M., Chen S., Wu Q. (2019). Avicularin inhibits cell proliferation and induces cell apoptosis in cutaneous squamous cell carcinoma. Exp. Ther. Med..

[25] Zhu X., Ouyang W., Miao J., Xiong P., Feng K., Li M., Cao Y., Xiao H. (2017). Dietary Avicularin Alleviated Type 2 Diabetes in Mice. FASEB J..

[26] Luo C., Yang H., Tang C., Yao G., Kong L., He H., Zhou Y. (2015). Kaempferol alleviates insulin resistance via hepatic IKK/NF-κB signal in type 2 diabetic rats. Int. Immunopharmacol..

[27] de Araújo E., Guerra G., Araújo D., de Araújo A., Fernandes J., Júnior R.D.A., da Silva V., de Carvalho T., Ferreira L., Zucolotto S. (2018). Gastroprotective and Antioxidant Activity of *Kalanchoe brasiliensis* and *Kalanchoe pinnata* Leaf Juices against Indomethacin and Ethanol-Induced Gastric Lesions in Rats. Int. J. Mol. Sci..

[28] Al-Numair K.S., Chandramohan G., Veeramani C., Alsaif M.A. (2015). Ameliorative effect of kaempferol, a flavonoid, on oxidative stress in streptozotocin-induced diabetic rats. Redox Rep..

[29] Suh K.S., Choi E.-M., Kwon M., Chon S., Oh S., Woo J.-T., Kim S.W., Kim J.-W., Kim Y.S. (2009). Kaempferol attenuates 2-deoxy-d-ribose-induced oxidative cell damage in MC3T3-E1 osteoblastic cells. Biol. Pharm. Bull..

[30] Palacz-Wrobel M., Borkowska P., Paul-Samojedny M., Kowalczyk M., Fila-Danilow A., Suchanek-Raif R., Kowalski J. (2017). Effect of apigenin, kaempferol and resveratrol on the gene expression and protein secretion of tumor necrosis factor alpha (TNF-α) and interleukin-10 (IL-10) in RAW-264.7 macrophages. Biomed. Pharmacother..

[31] Powers S.K., Ji L.L., Kavazis A.N., Jackson M.J. (2011). Reactive oxygen species: Impact on skeletal muscle. Compr. Physiol..

[32] Quintana-Cabrera R., Bolaños J.P. (2013). Glutathione and γ-glutamylcysteine in hydrogen peroxide detoxification. Methods Enzym..

[33] Asmat U., Abad K., Ismail K. (2016). Diabetes mellitus and oxidative stress—A concise review. Saudi Pharm. J..

[34] Pham-Huy L.A., He H., Pham-Huy C. (2008). Free radicals, antioxidants in disease and health. Int. J. Biomed. Sci..

[35] Steinbacher P., Eckl P. (2015). Impact of oxidative stress on exercising skeletal muscle. Biomolecules.

[36] Le Moal E., Pialoux V., Juban G., Groussard C., Zouhal H., Chazaud B., Mounier R. (2017). Redox Control of Skeletal Muscle Regeneration. Antioxid. Redox Signal..

[37] Kozakowska M., Pietraszek-Gremplewicz K., Jozkowicz A., Dulak J. (2015). The role of oxidative stress in skeletal muscle injury and regeneration: Focus on antioxidant enzymes. J. Muscle Res. Cell Motil..

[38] Kalyanaraman B. (2013). Teaching the basics of redox biology to medical and graduate students: Oxidants, antioxidants and disease mechanisms. Redox Biol..

[39] Chukwunonso Obi B., Chinwuba Okoye T., Okpashi V.E., Nonye Igwe C., Olisah Alumanah E. (2016). Comparative study of the antioxidant effects of metformin, glibenclamide, and repaglinide in alloxan-induced diabetic rats. J. Diabetes Res..

[40] Menon N., Sparks J., Omoruyi F.O. (2016). Oxidative Stress Parameters and Erythrocyte Membrane Adenosine Triphosphatase Activities in Streptozotocin-induced Diabetic Rats Administered Aqueous Preparation of *Kalanchoe pinnata* Leaves. Pharmacogn. Res..

[41] Phung C.D., Ezieme J.A., Turrens J.F. (1994). Hydrogen peroxide metabolism in skeletal muscle mitochondria. Arch. Biochem. Biophys..

[42] Chance B., Sies H., Boveris A. (1979). Hydroperoxide metabolism in mammalian organs. Physiol. Rev..

[43] Cohen G., Hochstein P. (1963). Glutathione Peroxidase: The Primary Agent for the Elimination of Hydrogen Peroxide in Erythrocytes. Biochemistry.

[44] Duranti G., Maldini M., Crognale D., Horner K., Dimauro I., Sabatini S., Ceci R. (2021). *Moringa oleifera* Leaf Extract Upregulates Nrf2/HO-1 Expression and Ameliorates Redox Status in C2C12 Skeletal Muscle Cells. Molecules.

[45] Alpert M. (2005). The diverse benefits of glutathione: A key antioxidant for reversing chronic illness. Altern. Complement. Ther..

[46] De Mattia G., Bravi M., Laurenti O., Cassone-Faldetta M., Armiento A., Ferri C., Balsano F. (1998). Influence of reduced glutathione infusion on glucose metabolism in patients with non-insulin-dependent diabetes mellitus. Metabolism.

[47] Njålsson R., Norgren S. (2005). Physiological and pathological aspects of GSH metabolism. Acta Paediatr. Int. J. Paediatr..

[48] Harlalka G., Patil C., Patil M. (2007). Protective effect of *Kalanchoe pinnata* pers. (Crassulaceae) on gentamicin-induced nephrotoxicity in rats. Indian J. Pharmacol..

[49] Aiyegoro O.A., Okoh A.I. (2010). Preliminary phytochemical screening and In vitro antioxidant activities of the aqueous extract of Helichrysum longifolium DC. BMC Complement. Altern. Med..

[50] Check Out This Tea Brewing Temperature Guide. https://www.thespruceeats.com/tea-brewing-temperature-guide-766367.

[51] Miranda A.F., Nette E.G., Khan S., Brockbank K., Schonberg M. (1978). Alteration of myoblast phenotype by dimethyl sulfoxide. Proc. Natl. Acad. Sci. USA.

[52] Klaren W.D., Rusyn I. (2018). High-Content Assay Multiplexing for Muscle Toxicity Screening in Human-Induced Pluripotent Stem Cell-Derived Skeletal Myoblasts. ASSAY Drug Dev. Technol..

[53] Wageesha N.D.A. (2014). ResearchGate. https://www.researchgate.net/post/What_must_be_the_maximum_final_DMSO_in_a_cell_culture_plate_24_well_plate_if_I_have_to_dissolve_my_compound_in_DMSO.

[54] Chien S.-W., Kuo D.-Y., Liao J.-M., Wang P.S., Yu C.-H. (2016). Growth Modulation of Diabetic Factors and Antidiabetic Drugs on Prostate Cancer Cell Lines. Chin. J. Physiol..

[55] Yue W., Zheng X., Lin Y., Yang C.S., Xu Q., Carpizo D., Huang H., DiPaola R.S., Tan X.-L. (2015). Metformin combined with aspirin significantly inhibit pancreatic cancer cell growth *in vitro* and *in vivo* by suppressing anti-apoptotic proteins Mcl-1 and Bcl-2. Oncotarget.

[56] Śmieszek A., Czyrek A., Basinska K., Trynda J., Skaradzińska A., Siudzińska A., Marędziak M., Marycz K. (2015). Effect of Metformin on Viability, Morphology, and Ultrastructure of Mouse Bone Marrow-Derived Multipotent Mesenchymal Stromal Cells and Balb/3T3 Embryonic Fibroblast Cell Line. BioMed Res. Int..

[57] Chen D., Xia D., Pan Z., Xu D., Zhou Y., Wu Y., Cai N., Tang Q., Wang C., Yan M. (2016). Metformin protects against apoptosis and senescence in nucleus pulposus cells and ameliorates disc degeneration in vivo. Cell Death Dis..

[58] He L., Wondisford F.E. (2015). Metformin action: Concentrations matter. Cell Metab..

[59] Biswas S.K., Chowdhury A., Das J., Karmakar U.K., Shill M.C. (2011). Assessment of cytotoxicity and antibacterial activities of ethanolic extracts of *Kalanchoe pinnata* Lin. (Family: Crassulacease) leaves and stems. Int. J. Pharm. Sci. Res..

[60] Chowdhury K., Huq M., Ali M., Huq I., Royhan M., Adnan M., Chy M.N.U., Kabir M.I., Auniq R.B.J., Uddin M.R. (2016). Antioxidant, cytotoxic and thrombolytic activity of leaves of *Kalanchoe pinnata* (LAM.) PERS. J. Pharmacogn. Phytochem..

[61] Waititu K., Jerono C., Kituku D., Nzuve M., Mambo F., Ngugi P., Mwethera P. (2018). Phytochemical Composition of *Kalanchoe pinnata* and Bidens pilosa Leaves Associated with Management of Diabetes. Biomed. Biotechnol..

[62] Cayman Chemical (2016). Superoxide Dismutase Assay Kit.

[63] Sinha A.K. (1972). Colorimetric assay of catalase. Anal. Biochem..

[64] Ellman G.L. (1959). Tissue sulfhydryl groups. Arch. Biochem. Biophys..

[65] Nguyen K., Sparks J., Omoruyi F.O. (2016). Investigation of the cytotoxicity, antioxidative and immune-modulatory effects of Ligusticum porteri (Osha) root extract on human peripheral blood lymphocytes. J. Integr. Med..

[66] Abcam (2016). Lipid Peroxidation (MDA) Assay Kit (Colorimetric/Fluorometric).

[67] R&D Systems Inc. (2013). TBARS Assay.

[68] (2017). Stanbio Total Protein. http://www.vitroscience.cl/pdf/stanbio/Proteina_Total_(LCR_Orina).pdf.

